# Hereditary transthyretin amyloidosis overview

**DOI:** 10.1007/s10072-020-04889-2

**Published:** 2020-11-14

**Authors:** Fiore Manganelli, Gian Maria Fabrizi, Marco Luigetti, Paola Mandich, Anna Mazzeo, Davide Pareyson

**Affiliations:** 1 Department of Neurosciences, Reproductive Sciences and Odontostomatology, University of Naples “Federico II”, Via S. Pansini, 5, 80131 Naples, Italy; 2 Section of Neurology, Department of Neuroscience, Biomedicine and Movement Sciences, University of Verona, Verona, Italy; 3 Fondazione Policlinico Universitario A. Gemelli. UOC Neurologia, Rome, Italy; 4 Dipartimento di Neuroscienze, Università Cattolica del Sacro Cuore, Rome, Italy; 5 Department of Neuroscience, Rehabilitation, Ophthalmology, Genetics and Maternal and Child Health (DINOGMI), University of Genoa, Genova, Italy; 6 IRCCS Policlinico San Martino, Genoa, Italy; 7 Unit of Neurology and Neuromuscular Diseases, Department of Clinical and Experimental Medicine, University of Messina, Messina, Italy; 8 Rare Neurodegenerative and Neurometabolic Diseases Unit, Department of Clinical Neurosciences, Fondazione IRCCS Istituto Neurologico Carlo Besta, Milan, Italy

**Keywords:** Transthyretin, TTR, ATTRv, Amyloidosis

## Abstract

Hereditary amyloidogenic transthyretin (ATTRv) amyloidosis is a rare autosomal dominantly inherited disorder caused by mutations in the transthyretin (TTR) gene. The pathogenetic model of ATTRv amyloidosis indicates that amyloidogenic, usually missense, mutations destabilize the native TTR favouring the dissociation of the tetramer into partially unfolded species that self-assemble into amyloid fibrils. Amyloid deposits and monomer-oligomer toxicity are the basis of multisystemic ATTRv clinical involvement. Peripheral nervous system (autonomic and somatic) and heart are the most affected sites. In the last decades, a better knowledge of pathomechanisms underlying the disease led to develop novel and promising drugs that are rapidly changing the natural history of ATTRv amyloidosis. Thus, clinicians face the challenge of timely diagnosis for addressing patients to appropriate treatment. As well, the progressive nature of ATTRv raises the issue of presymptomatic testing and risk management of carriers. The main aim of this review was to focus on what we know about ATTRv so far, from pathogenesis to clinical manifestations, diagnosis and hence patient’s monitoring and treatment, and from presymptomatic testing to management of carriers.

## Introduction

Hereditary amyloidogenic transthyretin (ATTRv; v for “variant”) amyloidosis is caused by mutations in the transthyretin (TTR) gene and is an autosomal dominantly inherited, debilitating, progressive and, if left untreated, fatal multisystem disorder. Prevalence of the disease is highly variable between the endemic and non-endemic countries and the global prevalence was estimated to be 10,186 persons (range 5526–38,468) [1].

The main aim of this review is to provide an overview on ATTRv amyloidosis from pathogenesis to clinical manifestations, diagnosis and hence patient’s monitoring and treatment and from presymptomatic testing to management of carriers.

### From protein to pathology

TTR is a homotetrameric protein with a backup carrier role of thyroxine (T4) in the plasma and cerebrospinal fluid, and by associating with the retinol-binding protein, it also mediates the transport of vitamin A. It is encoded by a small gene (chromosome 18q12.1) comprising only four exons. The monomers, formed after the cleavage of a 20 amino acid signal peptide, are composed of 127 amino acids arranged in eight antiparallel β-sheets; traditionally, the amino acid numbering refers to the mature protein. The homotetramer contains two T4-binding sites and binding of T4 contributes to its structural stability. Mutant and wild-type TTR may give rise in various tissues and organs to extracellular amyloid deposits formed by bundles of β-sheet fibrillar protein identified by apple-green birefringence under a polarized light microscope, after staining with Congo red, and by rigid unbranched fibrils 10–12 nm in diameter on electron microscopy [2].

TTR amyloidosis is a conformational disease and the pathologic protein aggregation is largely due to reduced folding stability. The pathogenetic model of ATTRv amyloidosis indicates that amyloidogenic, usually missense, mutations destabilize the native TTR favouring the dissociation of the tetramer into partially unfolded species that self-assemble into amyloid fibrils. The amyloidogenic potential of TTR variants correlates inversely with their thermodynamic stability.

Among over 130 mutations identified, the vast majority are pathogenic; a minority non-amyloidogenic, exceptional variants are protective in compound heterozygosity with pathogenic mutations.

Small molecule drugs such as tafamidis act as TTR stabilizers binding to unoccupied T4-binding sites. In ATTRv, amyloid deposits occur prevalently in the somatic and autonomic peripheral nervous system (PNS) and heart, though they may also involve kidneys, eyes, leptomeningeal vessels, joints and ligaments. This tissue specificity is elusive; endogenous factors such as glycosaminoglycans or the chemico-physical milieu might promote the amyloid deposition [3]. Due to the amyloidogenic potential of the wild-type monomers, ATTR amyloidosis may also be a non-hereditary (ATTRwt) disease, manifesting mainly as a cardiomyopathy in elderly men. Partial misfolding of the native wild-type TTR might be favoured by local chemico-physical factors [4]. An alternative pathogenetic model for ATTR amyloidosis involves mechano-enzymatic cleavages by trypsin and/or plasmin and biomechanical forces such as the shear stress by fluid flows that might be relevant particularly for cardiomyopathy [5].

The heterogeneity of the amyloidogenic pathways may explain the distinct biochemical composition of amyloid fibrils which are formed by C-terminal fragments (type A fibrils) in ATTRwt and in the majority of late-onset ATTRv and by full-length monomers (type B fibrils) in the early-onset V30M ATTRv amyloidosis. Such different compositions contribute to genotypic-phenotypic correlations and impact the sensitiveness of diagnostic procedures such as staining of the tissue biopsy by Congo red, which has a higher affinity for type B fibrils, or non-invasive techniques for amyloid imaging [6].

Amyloid fibrils may cause tissue damage by direct compression or obstruction as it is obvious for carpal tunnel syndrome, vitreous opacities and spinal canal stenosis. The PNS involvement is more likely caused by the neurotoxicity exerted by non-fibrillar oligomers and protofibrils [2]. Diffusible oligomers might bind to the lipid rafts of cell membranes, causing a calcium influx through voltage-gated calcium channels, and to receptors for advanced glycation end products thus interfering with the MAP kinase signalling and inducing endoplasmic-reticulum stress and apoptosis [4].

In ATTRv amyloidosis, the polyneuropathy has a length-dependent axonal pattern and affects variably both the large and small myelinated as well non-myelinated small fibres. In bioptic specimens, amyloid deposits predominate in the endoneurial blood vessels; deposits and axonal loss have an asymmetric distribution between and within the fascicles. Ultrastructural changes of the endothelial indicate a microangiopathy with a disruption of the blood-nerve barrier which may allow the entry of circulating TTR into the endoneurial space [7]. In the early-onset ATTR-V30M, which manifests with a predominant loss of small fibres, non-myelinating Schwann cells adjacent to amyloid fibrils are distorted and atrophic suggesting a direct effect on cell membranes by the amyloid fibrils. In the late-onset ATTR-V30M, which discloses fewer amyloid deposits, the prevalent involvement of the large myelinated fibres could be rather caused by neurotoxic oligomers [7].

From pathology to symptoms

Amyloid deposits and monomers/oligomers toxicity are the basis of ATTRv clinical presentation. PNS (somatic and autonomic) and heart are the most affected sites. Therefore, peripheral sensory-motor neuropathy, dysautonomia and cardiomyopathy, often in combination, are the common phenotypes [8].

Clinical heterogeneity is only partly explained by genetic mutation differences. The most frequent mutation worldwide, V30M, has either early-onset (in the endemic areas in Portugal and Brazil, mean onset age 33 years; occasionally in other areas including Italy) or late-onset (in Sweden—mean onset age 60 years—in many Japanese cases and in non-endemic countries like Italy), for still unknown reasons [8–10].

Early-onset V30M ATTRv is characterized by a small-fibre neuropathy with neuropathic pain, other positive sensory symptoms, algo-thermal sensory loss at distal limbs; only later touch and deep sensory loss and motor involvement become evident, with a length-dependent distal-to-proximal progression [11]. Autonomic symptoms are common and relevant and consist of erectile dysfunction, orthostatic hypotension, reduced sweating, dry eye and mouth, pupillary changes, bladder abnormalities and gastrointestinal dysmotility—causing early satiety, gastric distension, recurrent nausea and vomiting, diarrhea and/or stipsis [12]. Gastrointestinal dysfunction, also related to direct amyloid infiltration, may play a role in weight loss, a frequent feature of all ATTRv forms [8]. Heart involvement is mainly characterized by arrhythmias, bundle and atrio-ventricular blocks, more rarely sinoatrial blocks, often needing pacemaker positioning [11]. Genders are equally affected and family history is often informative [11].

Late-onset V30M ATTRv has a different clinical picture: the neuropathy affects small and large fibres since the beginning, with sensory loss to all modalities, early muscle wasting and weakness starting from distal sites [8, 10]. Autonomic involvement is often subtle and undetected if not investigated. Heart dysfunction consists in a hypertrophic infiltrative cardiomyopathy, with preserved ejection fraction, which may be severe and progressive [13]. Given later onset and age-dependent penetrance, family history is frequently negative. Males are more commonly affected than females with a 2–3:1 ratio.

The disease course, lethal if left untreated after a mean of 7–10 years, is more rapid in the late-onset variety [8]. The difference between the two forms is likely explained by different types of amyloid deposits: full-length TTR forming regularly disposed fibril with high Congo red affinity in early-onset V30M (type B); a mixture of full-length and cleaved TTR fragments, with irregularly arranged fibrils showing low Congo Red affinity in late-onset V30M (type A) [14].

In non-endemic countries, including Italy, there are many other mutations, some (e.g. *E89Q*, *F64L*) are also frequent, sharing similarities with the late-onset V30M type, including onset age. *T49A* and *E89Q* show fast progression; *F64L* is relatively less rapid; *I68L* is predominantly cardiopathic [9, 15].

Several patients show clinical and/or electrophysiological features of bilateral carpal tunnel syndrome, related to amyloid deposits in the transverse carpal ligament, sometimes preceding by years polyneuropathy onset [16]. Less common atypical phenotypes are large-fibre neuropathy with sensory ataxia, preeminent motor involvement, non-length-dependent pattern with early cranial nerve involvement or upper limb predominance [8]. The progressive neurological impairment leads the untreated patient, who initially has retained walking ability [familial amyloid polyneuropathy (FAP) stage 1], to need assistance for walking (FAP stage 2) and later lose walking ability (FAP stage 3, chairbound/bedridden) [17].

Nephropathy rarely occurs with proteinuria, renal failure, recurrent urinary infections [18]. Ocular involvement is characterized by intraocular amyloid deposits related to the TTR production by the retinal epithelium, causing vitreous opacities, glaucoma and retinal amyloid angiopathy [19]. Few mutations are specifically associated with the exceptional oculo-leptomeningeal disease form [20]. TTR production by choroid plexuses is the basis of the rare leptomeningeal variant, with amyloid deposition starting from leptomeningeal vessels and involving—in a centripetal progression along vessels—the brain parenchyma, and manifesting with seizures, hemorrhagic strokes, focal neurological episodes, dementia, ataxia, hydrocephalus, siderosis, calcifications and leptomeningeal enhancement [21].

### From neuropathy to diagnosis

**Fig. 1:**
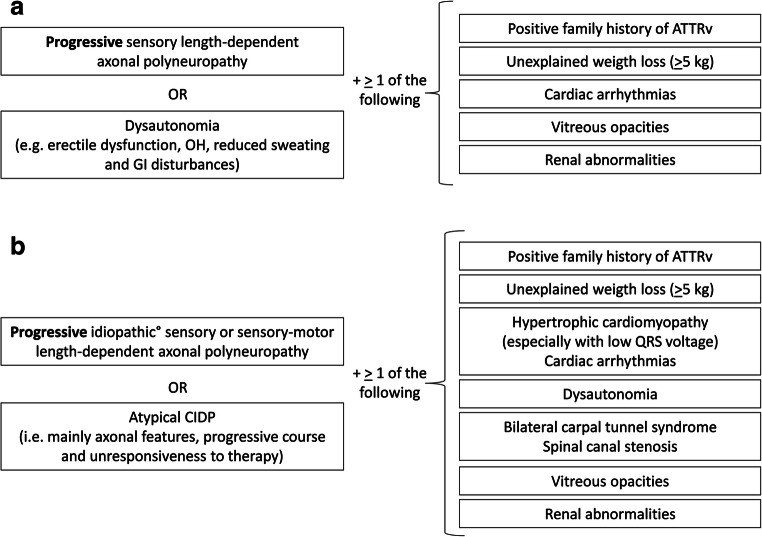
Suspicion index for diagnosis of ATTRv amyloidosis with PN [adapted from Adams et al. 2019 [8]]. **a** In early-onset phenotype. **b** In late-onset phenotype. ATTRv, hereditary amyloidogenic transthyretin amyloidosis; CIDP, chronic inflammatory demyelinating polyradiculoneuropathy, GI gastrointestinal, OH orthostatic hypotension. Screening test for more common peripheral neuropathy negative

PNS involvement is the presenting complaint in most cases of ATTRv. The main challenge for clinicians in evaluating a neuropathic patient is when to suspect ATTRv in order to catch early the diagnosis. In regions where ATTRv amyloidosis is non-endemic, diagnosis can be delayed by 3–4 years [8]. The importance of early diagnosis is enormously increased after recent availability of innovative disease-modifying therapies (see section “Current and emerging therapies”). Thus, bearing in mind the importance of early diagnosis of disease, some clinical, laboratory and instrumental features may raise the suspicion of ATTRv amyloidosis (Fig. 1).

Overall, ATTRv neuropathy is a progressive length-dependent axonal polyneuropathy involving small (sensory and autonomic) and large (sensory and motor) nerve fibres [8, 22].

A preferential and early involvement of small fibres is typical of early-onset V30M ATTRv phenotype [22]. The initial small fibre neuropathy progresses to the involvement of larger fibres (sensory and motor) leading to accumulation of disability and ultimately to death [8].

On the other side, in late-onset ATTRv phenotypes (V30M and most of non-V30M), pathological evidence demonstrates early involvement of all types of nerve fibres even though the impairment of the largest fibres usually overcomes the smallest ones [7, 22].

Therefore, clinicians may face a first scenario including a neuropathic patient complaining of dysautonomia. In such a case, though alternative diagnosis may be considered, genetic testing for ATTRv amyloidosis should not be delayed [8, 23, 24].

Another scenario, more challenging for clinicians and more common in non-endemic areas, includes sporadic patients with late-onset phenotype that present with a sensory or sensory-motor length-dependent axonal polyneuropathy.

In such a case, electrophysiological features may be similar to several other neuropathies including those dysmetabolic (e.g. diabetes mellitus), toxic, nutritional, paraneoplastic, infectious or late-onset Charcot-Marie-Tooth disease. Laboratory screening test for peripheral neuropathy is needed even though comorbidities as diabetes may coexist with ATTRv amyloidosis [25, 26].

The most striking feature of ATTRv neuropathy in differential diagnosis is its progressive course. On average, patients with early-onset form switch from FAP-1 to FAP-2 stage of disease in 5.6 years and from FAP-2 to FAP-3 in 4.8 years. In patients with late-onset form, the progression is even faster and requires 2–4 years for switching from FAP-1 to FAP-2 and 2–3 years from FAP-2 to FAP-3 [27, 28].

This is also important in differential diagnosis from chronic inflammatory demyelinating polyradiculoneuropathy (CIDP) that represents the most common misdiagnosis [29–32]. This comes from the possibility to find in ATTRv patients nerve conduction slowing in range of demyelination [33]. Actually, demyelination occurs in ATTRv neuropathy and myelin alterations are usually in close contact with TTR deposits [8, 30, 31, 34]. Importantly, the chance to detect amyloid deposits in peripheral nerves seems to be related to disease duration in both early- and late-onset ATTRv [8, 22, 34], and accordingly, in late stage of disease, sural nerve findings usually reveal frequent amyloid deposits, conspicuous axonal loss and myelin abnormalities [8, 30, 31, 34]. As well, skin biopsies show, in early stage of disease, a loss of nerve fibres but none or minimal deposition of amyloid with respect to more advanced stage of ATTRv amyloidosis [35, 36].

Therefore, ATTRv patients, usually in more advanced stage of disease, may be mistaken for CIDP, but a careful reading of electrophysiological findings reveals that slow nerve conduction velocity is associated with axonal loss and accordingly with a severe reduction of compound muscle action potential amplitudes [30–32]. Unresponsiveness to therapy (e.g. immunoglobulin) may further raise the suspicion of ATTRv.

Another challenging misdiagnosis encompasses paraproteinemic neuropathies, i.e. amyloid light-chain (AL) amyloidosis and POEMS syndrome [37, 38], that show clinical and electrophysiological similarities with ATTRv neuropathy for coexistence of axonal and demyelinating features as well for multisystem involvement (e.g. heart in AL amyloidosis). Since a paraproteinemia of unknown significance may coexist with ATTRv amyloidosis [38], a hematological evaluation is needed to rule out plasmacellular dyscrasia. In addition, scintigraphy to detect cardiac uptake of bone tracers such as ^99m^Tc-DPD, ^99m^Tc-HMDP, or ^99m^Tc-PYP [39] may be useful in differentiating between monoclonal immunoglobulin light chain and TTR-related cardiac amyloidosis. The possibility of a low sensitivity for specific mutations (e.g. *Phe64Leu*) should be considered [40]. On the other hand, many studies confirm that VEGF levels could be useful for differentiating POEMS syndrome from amyloidosis [37].

### After the diagnosis

Once the diagnosis of ATTRv amyloidosis has been reached, additional investigations are required to evaluate the extent and severity of organ involvement.

To monitor the polyneuropathy disability, useful tools in clinical practice include the FAP staging system and the polyneuropathy disability (PND) score [17]. However, these clinical scales provide only a generic indicator of overall disease status and are not sensitive to track disease progression in the short-term period. Hence, to better evaluate all the aspects of the polyneuropathy, clinical trials have tested different neuropathy impairment score (NIS)-based measures, which provide a better chance to detect a treatment effect. Nevertheless, the NIS score [41], a clinical compound score based on examination of muscle weakness, sensory loss and stretch reflexes in the limbs, and its subset, the NIS-lower limbs (NIS-LL) score, have shown some limitations, requiring gradual modifications.

In a trial published in 2013, the NIS + 7, which combines clinical assessment with seven electrophysiological tests, was used to better characterize and quantify neuropathic impairment [41]. In recent trials, run by Alnylam [42, 43] and by Ionis [44], two different variants of the modified NIS + 7 (mNIS + 7) were used. The mNIS + 7_Alnylam_ and the mNIS + 7_Ionis_, specifically designed to assess disease impairment and progression in hATTR amyloidosis clinical trials, better quantify sensory abnormalities over the whole body, nerve conduction abnormalities and autonomic function [41].

Other useful clinical scales include the Composite Autonomic Symptom Scale-31 (COMPASS-31) questionnaire and the Compound Autonomic Dysfunction Test (CADT) questionnaire for assessment of autonomic symptoms; the Rasch-built Overall Disability Scale (R-ODS) survey for evaluation of activities of daily living; Charcot-Marie-Tooth Neuropathy scale (CMTNS) and its clinical component CMT Examination Score (CMTES) for monitoring neuropathy progression; the Norfolk Quality of Life-Diabetic Neuropathy (QoL-DN) questionnaire to estimate quality of life; the 6-min walking test, the 10-m walking test and handgrip strength test (dynamometer) to assess specific motor function [41–45].

Traditional nerve conduction studies are performed to monitor the evolution and the severity of a peripheral neuropathy [46]. Even if skin biopsy still remains the gold standard for the diagnosis of a small fibre neuropathy, evaluation of sudomotor function via electrochemical skin conductance [47], as well as measurement of heart rate variability [48], and testing for orthostatic hypotension can all be useful to investigate an autonomic neuropathy. Sudoscan also proved to be a good tool for monitoring disease progression in late-onset hATTR [47].

Cardiac investigations in ATTRv amyloidosis are mainly aimed at detecting a possible infiltrative (hypertrophic) cardiomyopathy and, above all, any potential serious conduction disorders that may require the implantation of a prophylactic pacemaker to decrease the risk of sudden death. Useful investigations for cardiac assessment include echocardiogram, 24-h Holter monitoring and echocardiography with strain imaging; cardiac magnetic resonance imaging (MRI) and intracardiac electrophysiological studies, even if not always available, are useful when necessary [49].

Cardiac serum biomarkers, specifically brain natriuretic peptide (BNP) or its N-terminal prohormone (NT-proBNP) and cardiac troponins (T or I), are useful and have a prognostic value in amyloid cardiomyopathy [49]. NT-proBNP plasma levels are abnormal even in the early stages of cardiac amyloid infiltration and correlate with left ventricular mass (evaluated on cardiac MRI) and with late gadolinium enhancement, suggesting their utility as a measure of cardiac amyloidosis severity [49]. In addition, high levels of troponin are observed in the most severe forms or in advanced stages of the disease [49].

Once the genetic diagnosis is confirmed, an ophthalmological assessment should be carried out as well. When ocular manifestations are present, the frequency of ophthalmological evaluations varies depending on severity of eye involvement and should include measurement of visual acuity and of intraocular pressure, Schirmer test, ocular fundus and slit-lamp examination [50].

Renal evaluation is also crucial, and usually based on measurement of serum creatinine, proteinuria and microalbuminuria, and on the estimated glomerular filtration rate (eGFR) [46].

Finally, a modified version of the body mass index (mBMI) that corrects for the effect of hypoalbuminaemia provides a marker of nutritional status, which is, to some extent, influenced by the duration and severity of gastrointestinal symptoms and malabsorption in hATTR patients [51].

#### From presymptomatic testing to carriers

Protocols for pre-symptomatic genetic testing (PST) of late-onset inherited disorders are available since many years, including that for genetic testing and management of individuals at risk for ATTRv [52–54].

The main purpose of all these protocols is to provide with participants a multidisciplinary team and whole information to protect them against psychosocial consequences of the test results. All PST protocols aim to combine the respect for the autonomy with the maximum benefit, supporting the at-risk individual in the decision-making process and helping her/him to cope with the results.

All relatives of patients with ATTRv should be considered as possible carriers of the familial mutation and, if they are willing to undergo genetic testing, they should be directed to an expert multidisciplinary team. The whole process should involve teams with expertise in genetic counselling, in providing accurate interpretation of molecular results and in disease-specific management and follow-up. Due to the possible psychological impact of the test results, a psychologist with expertise in genetic counselling must be available from each team.

Although all adult patient’s relatives can undergo ATTRv testing, the potential benefits of PST are greater for siblings than for the offspring. In fact, siblings, especially those that are close to the predicted age of disease onset (PADO) [55], are at higher risk for developing clinical disease in the immediate future and deserve the highest priority.

All adult at-risk individuals who may wish to take the test should be given updated and relevant information in order to make an informed voluntary decision. Pre-test counselling should include information not only about the entire testing process, but also about the post-test follow-up.

In general, a minimum interval (e.g. 1 month) between the pre-test counselling session and the decision whether to take the test is habitually recommended to give the person enough time in order to make an informed and autonomous decision. The decision to take the test is the unique choice of the person concerned.

Disclosure of PST results should be done preferably within 4 weeks from blood sample collection. Nevertheless, the proband should have the choice to ask for more time delay before receiving the results or also decide not to be given the results at all [56].

Test result should be given in person to the individual who requested the PST. As a rule, the counselling team should not communicate any information concerning the test and its results to third parties without the explicit permission of the person tested.

If the genetic analysis is positive, the multidisciplinary team must address the subject to the appropriate follow-up program, already discussed before the test, and based on the family mutation and the his/her actual age.

Compared with PST for other late-onset untreatable diseases, the protocol for ATTRv genetic testing has undergone some changes to make it more up-to-date. In fact, in the last years, the ATTRv therapeutic scenario has dramatically changed thanks to the availability of new drugs which are able to treat the disease. Since all therapies are maximally effective in the early stages, the request for PST has largely increased as the possibility to access a therapy balances or outweighs the risk of the psychological consequences of a positive test result. In fact, nowadays, clinicians can offer individuals with positive test result a close monitoring [55], prompting treatment start as soon as minor, but clinically meaningful disease signs, are detected. The PST protocol should be regularly updated in order to offer a flexible approach in accordance with drug discoveries. Attempts to identify new early biomarkers of the progression from an asymptomatic status to the appearance of the first signs of the diseases are currently under investigation [57]. Therefore, clinicians must be prepared to adapt PST to rapid changes in the ATTRv therapeutic landscape to provide the best of care for presymptomatic individuals.

### Current and emerging therapies

Elucidation in molecular pathogenic mechanisms and progress in pharmacological technologies led to an unexpected therapeutic revolution in ATTRv amyloidosis. A wide spectrum of targeted therapies already obtained market access and some others are close to achieve it [58, 59].

Until recently drugs, symptomatic relief and orthotopic liver transplant were the only options for ATTRv with the most favourable results in early-onset V30M patients [60].

Recently, new agents have been developed to suppress the production of both amyloidogenic wt and TTRv as well as fibril formation. The landscape of current pharmaceutical approaches for ATTRv includes TTR stabilizer, TTR silencers and TTR disruptors.

#### TTR stabilizer

Tafamidis meglumine (Vyndaqel, Pfizer) was the first specific drug approved for stage 1 ATTRv-PN on the basis of an 18-month double-blind placebo-controlled study followed by an open label extension [61]. Later on, several clinical trials supported these findings with newsworthy results in V30M patients and early disease stages [62]. Tafamidis was generally well tolerated, even for long periods [63] and induced reduction of all-cause mortality and cardiovascular related hospitalizations [64]. Tafamidis is now approved in the USA for ATTR cardiomyopathy (CM).

Diflunisal, a non-steroidal anti-inflammatory drug, reduced the progression of neuropathy irrespective of mutation and severity of disease at baseline [65]. Safety and efficacy of diflunisal were reported in respect to neurological and cardiac functions [66], and to autonomic dysfunction [67]. Diflunisal is not approved for ATTRv and can be used only “off-label”.

Epigallocatechin-3-gallate (EGCG), the major catechin found in green tea, appears to be able to prevent fibril formation “in vitro” and in cell culture and to disrupt pre-formed fibrils “in vitro” and in animal models [68]. Two studies reported a positive result of green tea consumption in wt- and ATTRv patients with cardiomyopathy [69, 70].

Tolcapone stabilizes three leptomeningeal TTR variants, and because it crosses the blood-brain barrier, it has been proposed as a therapy for leptomeningeal amyloidosis [71].

AG10 was well tolerated and induced a significant stabilization of TTR in ATTR-CM [72].

Two-phase three studies, in ATTR-CM and ATTRv-PN, respectively, are still ongoing (ATTRIBUTE-CM, ClinicalTrials.gov Identifier NCT03860935; ATTRibute-PN, ClinicalTrials.gov Identifier: NCT04418024).

#### TTR silencers and genome editing

TTR gene silencing therapy with small interfering RNA (siRNA) or antisense oligonucleotide (ASO) provided a therapeutic revolution, showing evidence that disease progression can be slowed, and perhaps reversed [42–44].

Both patisiran (a siRNA) and inotersen (a second-generation ASO) are approved by the EMA and FDA for ATTRv-PN.

A phase 1/2 study with a new GalNac-conjugated ASO (ION-682884) has been initiated (ClinicalTrials.gov Identifier: NCT03728634), and a phase 3 is ongoing.

A phase 3 study with vutrisiran, a new siRNA, is also ongoing. CinicalTrials.gov Identifier: NCT03759379.

CRISPR/Cas9-mediated genome editing approach has been found effective in mouse and rat models [73]. A phase 1 ascending-dose trial is going to be planned.

#### Fibril disruptors

Doxycycline and tauroursodeoxycholic acid (TUDCA) are not approved for ATTRv, although they both showed interesting results in experimental studies and combination of oral doxycycline and TUDCA stabilizes the disease for at least 1 year in ATTRv and wt [74, 75]. A phase 3 study of Doxy/TUDCA in cardiac amyloidosis is ongoing. ClinicalTrials.gov Identifier: NCT03481972.

#### Monoclonal antibodies

Several monoclonal antibodies against TTR epitopes have been tested “in vitro” as potential new drugs [76].

Dezamizumab is a fully humanized monoclonal IgG1 anti-SAP antibody that triggers immunotherapeutic clearance of amyloid. Amyloid load reduction was reported in the liver, spleen, and kidney following his administration in AL and ATTR amyloidosis [77].

A phase 1 clinical trial with PRX004 is undergoing. ClinicalTrials.gov Identifier: NCT03336580.

## Conclusion

Improved knowledge of pathogenetic mechanisms and the development of efficacious drugs are rapidly changing the history of ATTRv amyloidosis. Thus, the progressive nature of ATTRv emphasizes the need for timely diagnosis and intervention in patients and raises the future challenge of when treating the carriers.
